# Technological Aptitude and Applications of *Leuconostoc mesenteroides* Bioactive Strains Isolated from Algerian Raw Camel Milk

**DOI:** 10.1155/2013/418132

**Published:** 2013-12-11

**Authors:** Zineb Benmechernene, Hanane Fatma Chentouf, Bellil Yahia, Ghazi Fatima, Marcos Quintela-Baluja, Pilar Calo-Mata, Jorge Barros-Velázquez

**Affiliations:** ^1^Laboratory of Applied Microbiology, Department of Biology, Faculty of Sciences, Oran University, B.P. 16, Es-Senia, 31100 Oran, Algeria; ^2^Department of Analytical Chemistry, Nutrition and Food Science, School of Veterinary Sciences/College of Biotechnology, University of Santiago de Compostela, Rúa Carballo Calero s/n, Campus Lugo, 27002 Lugo, Spain

## Abstract

Two strains (B7 and Z8) of the *Leuconostoc mesenteroides* subspecies *mesenteroides* that were isolated from Algerian camel milk from an initial pool of 13 strains and demonstrated a high ability to inhibit the growth of *Listeria* spp. were selected and characterised at the phenotypic and genotypic levels. Probiotic profiling and inhibition spectra against food borne pathogens in mixed cultures were also investigated. The bacteriocin produced by *L. mesenteroides* strain B7 was identified as leucocin B by specific PCR. *In vitro* studies demonstrated that both *Leuconostoc mesenteroides* strains exhibited a marked probiotic profile, showing high survival at low pH (2-3 and 4) in the presence of 0.5%, 1%, and 2% of bile salts and at pH 3 in the presence of 3 mg/mL pepsin. Susceptibility testing against antimicrobial agents was also performed for both strains. When tested in a mixed culture with *Listeria innocua*, *Listeria ivanovii*, or *Staphylococcus aureus*, strain B7 reduced the numbers of these species by 1.87, 1.78, and 1.38 log units, respectively. Consequently, these two strains were found to possess good probiotic properties *in vitro* and a high capacity for *Listeria* spp. inhibition in mixed cultures. Therefore, these strains have a favourable technological aptitude and a potential application as novel probiotic starters.

## 1. Introduction

Camel milk has antimicrobial activity and a good conservation aptitude. Barbour et al. [1] reported that camel milk inhibits some pathogenic bacteria because of several protective proteins found in the milk, including lysozymes, lactoperoxidase, lactoferrin, immunoglobulin, and vitamin C. For these reasons, Yagil et al. [2] support that pasteurisation is not essential for camel milk if the camels are in good health. Camel milk composition is less stable than milk from other animals. These variations can be caused by many factors, such as geographical lactation, alimentary conditions, and the breed and age of the camel [3]. Lactic acid bacteria (LAB) from cow and goat milk have been well studied for both antimicrobial activity and bacteriocin production [4–6]. However, few studies have been conducted on the isolation and characterisation of LAB from camel milk [7–9] or on the antimicrobial activity [10–12].

Several LAB species produce a wide variety of antimicrobials that can be used for food preservation. In a number of instances, the inhibitory activity of LAB could be attributed to metabolic end products such as hydrogen peroxide, diacetyl and organic acids, and bacteriocin [13]. Currently, LAB include (13) different bacterial genera:* Lactobacillus*, *Leuconostoc*,* Lactococcus, Enterococcus, Streptococcus, Pediococcus, Carnobacterium, Oenococcus, Weissella, Aerococcus, Tetragenococcus, Vagococcus*, and* Bifidobacterium. *These bacteria are used primarily as starters in fermented food products where they can develop certain organoleptic characteristics and increase the time of conservation [14–17].

LAB exhibit probiotic properties because these bacteria are normal flora in gastrointestinal tract [18] and have no harmful effects [19]. Moreover, the addition of antagonistic bacterial preparations as protective cultures is another approach for biopreservation [20]. In addition, possible microbial interactions, either beneficial (cooperation) or unfavourable (inhibition), can be achieved in mixed strain cultures. These mixed cultures are commonly used as starter cultures in dairy manufacturing because of the acid production, growth rate, proteolytic activity, bacteriocin production and sensitivity, aroma production, and phage sensitivity of these cultures [21]. However, these technologies can be limiting for commercial application because of the negative impacts or the low reproducibility percentage of the response [22]. For these reasons, the characterisation of LAB as probiotic cultures should respond to several criteria, such as the ability to survive the specific conditions of the gastrointestinal tract, including low pH, proteolytic enzymes, and bile salt concentrations [23].

Because these bacteria are meant to be used as protective cultures, the inhibition pattern, safety, and functional and technological properties of these bacteria should also be examined.

Few studies have reported the high potential of using* Leuconostoc* as a probiotic strain. Kekkonen et al. [24] showed that the use of* Leuconostoc* as probiotic strain in inducing cytokines was better than that of the probiotic* Lactobacillus* strain that is presently in clinical use. In addition, Allameh et al. [18] reported for the first time the use of* Leuconostoc mesenteroides* isolated from the intestine of snakehead fish as a potentially new probiotic for aquaculture systems for the development of fish production. Moreover,* Leuconostoc lactis* has been shown to have a potential for use as a single starter culture in Khanoon-jeen production and could reduce the microbial risk and fermentation processing time [25]. Additionally, *Leuconostoc* strains have potential as protective cultures for vacuum packed meat products [26], and the bacteriocins of* Leuconostoc* can be used as protective agents in combination with another starter culture in fermented meat [27]. However, the use of* Leuconostoc mesenteroides* isolated from camel milk as either a probiotic strain or a protective culture has never been reported. Therefore, we isolated and identified* Leuconostoc* strains from camel milk as new probiotic or protective cultures for the first time.

## 2. Materials and Methods

### 2.1. Raw Camel Milk Sampling

Thirteen* Leuconostoc* strains were isolated from four different camel milk samples, which were collected from two different Saharan areas (Nâama and Béchar) in South Western Algeria. The first two samples were collected from two camels (*Camelus dromados*) in Nâama that differed in age (10 and 15 years old) and colour (grey and black) but had the same lactation period (March, 2011). The diet of the camels was based on natural Saharan plants, such as Drinn (*Arstide pungens*). Samples from Béchar were collected from brown camels aged less than 10 years in the arid Abadla region in the period 2009–2011. Sampling was performed under aseptic conditions by washing the teats with warm water containing 2% bleach and collecting the milk in sterile bottles after hand washing with diluted alcohol. Samples were maintained at 4°C and were transported in iceboxes to the laboratory for analysis.

### 2.2. *Leuconostoc* Isolation

The bioactive* Leuconostoc* considered in this work were isolated from the raw camel milk as described above. All strains were stored at −80°C and were maintained in reconstituted skimmed milk containing 30% (w/v) glycerol. All strains were cultured in MRS broth (Liofilchem, Teramo, Italy) at 30°C for 24 h and were then seeded onto MRS agar (Liofilchem) to obtain single colonies. The wild type and reference* Leuconostoc* strains used in this study belonged to the collection of our laboratory.

Based on the results obtained from the inhibitory assays, two bioactive leuconostocs strains (B7 and Z8) were selected and subjected to phenotypic and morphological characterisation based on the following criteria: CO_2_ production, growth at different temperatures (4°C, 15°C, 30°C, 37°C, and 45°C), growth at different pH (4.8 and 6.8), and growth at different NaCl concentrations (3% and 6.5%). Additionally, all strains were subjected to the following biochemical tests to differentiate between* Leuconostoc* and *lactobacilli*: dextran production on MSE medium [28], arginine hydrolysis on M16BCP medium (Oxoid Ltd., London, UK), and citric acid degradation on Kempler and McKay solid medium. Carbohydrate fermentation was tested on MRS supplemented with bromocresol purple as a pH indicator using the following sugars to differentiate the subspecies of *Leuconostocs*: arabinose, maltose, rhamnose, esculin, manitol, sorbitol, galactose, lactose, fructose, glucose, sucrose, and xylose. All strains were phenotypically identified as belonging to the* Leuconostoc* genus based on the following criteria: ovoid shape, Gram-positivity, catalase negativity, vancomycin-resistance, production of gas from glucose, lack of arginine hydrolysis, and fermentation profiles.

### 2.3. Genetic Identification of Bioactive *Leuconostoc* Strains

A fragment of the 16S rRNA gene of the two bioactive strains was amplified by PCR using the universal primer pair p8FPL (forward: 5′-AGTTTGATCCTGGCTCAG-3′) and p806R (reverse: 5′-GGACTACCAGGGTATCTAAT-3′) [29]. The assays comprised 100 ng of template DNA, 25 *μ*L of a master mix (BioMix, Bioline, London, UK) (this included the reaction buffer, dNTPs, and magnesium chloride), Taq DNA polymerase, 25 pmol of each oligonucleotide primer, and double-distilled water to achieve a final volume of 50 *μ*L. Amplification conditions were as follows: denaturing at 94°C for 7 min., 35 cycles of denaturation (94°C for 60 sec.), annealing (55°C for 60 sec.), extension (72°C for 60 sec.), and a final extension at 72°C for 15 min. The PCR was performed as described by Böhme et al. [30].

The two PCR products were then sequenced using the same primers used for PCR. The sequences were analysed with Chromas software (Griffith University, Queensland, Australia) and aligned using Clustal-X software [31, 32]. Following alignment, these sequences were identified by searching for sequence homology among published reference sequences using the web BLAST tool (National Center for Biotechnology Information (NCBI, http://blast.ncbi.nlm.nih.gov/) [33]. Homologies higher than 99% with respect to a strain type were considered acceptable identifications.

### 2.4. Genetic Identification of Bacteriocin Produced by *Leuconostoc* mesenteroides Strains

The bacteriocins produced by* L. mesenteroides* strains were identified by PCR using the primers described by Xiraphi et al. [34]. The detection of the following bacteriocins: mestenterocin B, mesenterocin Y, leucoccin A, leucoccin B, and leucocin A-TAF was carried out using the following primers: mesB, mesY, lcnA, lcnB, and lcnA-TAF, respectively. The reaction conditions were as described by Xiraphi et al. [34]. The nucleotide sequencing of the bacteriocin gene was performed as described above.

### 2.5. Probiotic Evaluation of Bioactive *L. mesenteroides* Strains

#### 2.5.1. Inhibition Spectra of *L. mesenteroides* Strains against Indicator Microorganisms

Preliminarily, all strains were tested for the ability to produce antimicrobial substances using the direct method described by Fleming et al. [35]. Inhibitory activity was investigated using the following indicator bacteria:* Staphylococcus aureus*: 43300 (Centre Hospitalier Universitaire, C.H.U Oran, Algeria),* Listeria innocua* (ATCC 33090), and* Listeria ivanovii* (ATCC 19119).

Alliquots of 80 *μ*L from 18 h cultures of 10^7^ CFU mL^−1^ *Leuconostoc* strains were spotted onto MRS agar using multipoint inoculators and were incubated at 30°C for 24 h [36]. Following the incubation, a semisolid Mueller Hinton (Oxoid) medium containing 100 *μ*L of 10^7^ CFU mL^−1^ of the indicator culture was poured as an overlay. All plates were then incubated at 37°C for 24 h and examined for inhibition zone formation. Inhibition was considered positive when the width of the clear inhibition halos was ≥0.5 cm.

#### 2.5.2. Detection of the Proteinaceous Nature of the Inhibitory Agent

The proteinaceous nature of the inhibitory substance was detected using an indirect method. In this method, a* Leuconostoc* strain was incubated for 18 h in MRS broth at 30°C and was then centrifuged at 8,000 rpm for 10 minutes. Then, 100 *μ*L of the supernatant (FCS) was inoculated on wells formed on solid MRS medium that were seeded by indicator strains, and the wells were incubated for 24 h to 48 h at 37°C. Colonies surrounded by a clear zone with a diameter greater than 2 mm in the layer of the indicator culture were considered positive. Several factors were eliminated to confirm the proteinous nature of the inhibitory substance, such as lactic acid using a buffered medium and the elimination of the effect of hydrogen peroxide by using indicator strains with catalase enzyme such as* S. aureus*,* Listeria innocua*, and* L. ivanovii*. In addition, proteolytic enzymes (trypsin-chymotrypsin) and heat treatments of the supernatant at different temperatures (75°C, 80°C, and 100°C) were used to identify the proteinaceous nature of the inhibitory substance.

#### 2.5.3. pH Tolerance

Bacterial cells from overnight MRS cultures were collected by centrifugation and were washed with sterile phosphate buffer saline, pH 8. Centrifugation and washing procedures were repeated three times. The bacterial cells were resuspended in sterile PBS adjusted to pH 2, 3, or 4 and were incubated at 37°C for 3 h. The viable bacterial counts were then determined in MRS agar.

#### 2.5.4. Bile Salt Tolerance and Bile Salt Hydrolyses

Bacterial cells from overnight MRS cultures were harvested by centrifugation, washed, and resuspended in PBS (pH 8) supplemented with 0.5%, 1.0%, or 2.0% (w/v) oxgall (Oxoid Ltd., England). Viable cell counts were determined in MRS agar after 4 h at 37°C.

For the bile salt hydrolysis assay, overnight bacterial cultures of each* L. mesenteroides* strain were streaked on MRS agar, supplemented with 0.5% (w/v) oxgall, and incubated for 24 and 48 h at 37°C. The bacterial hydrolysis of the bile salt was visualised as altered colony morphology compared with the control MRS plates.

#### 2.5.5. Resistance to Pepsin

Bacterial cells from overnight MRS cultures were collected, washed, and resuspended in PBS buffer (pH 2 and 3) supplemented with 3 mg/mL of pepsin. The resistance of the* L. mesenteroides* strains was determined by counting the initial viable cells in MRS agar after 3 h incubation at 37°C.

#### 2.5.6. Haemolytic Activity

An overnight culture of the* L. mesenteroides* strains was streaked in triplicates on Columbia agar plates containing 5% (w/v) human blood and incubated for 48 h at 30°C. Blood agar plates were examined for signs of *β*-haemolysis (clear zones around colonies), *α*-haemolysis (green zones around colonies), and *γ*-haemolysis (no zones around colonies).

#### 2.5.7. Antibiotic Sensitivity Test

The antibiotic susceptibility of the two strains of* L. mesenteroides* (B7 and Z8) was tested three times against 13 antibiotics using Bio-Rad discs (6 mm).* L. mesenteroides* strains were cultured in MRS broth at 30°C for 18 h and were then adjusted to a 0.5 McFarland scale and smeared homogeneously on MRS culture plate. Antibiotic discs were placed on the plates and incubated for 24 h at 37°C. The antibiotics included gentamycin (GM, 10 *μ*g), streptomycin (S, 10 *μ*g), amoxicillin (AMX, 25 *μ*g), tetracycline (TE, 30 *μ*g), chloramphenicol (C, 30 *μ*g), ampicillin (AM, 10 *μ*g), erythromycin (E, 15 *μ*g), cephalotin (CEF, 30 *μ*g), lincomycin (L, 15 *μ*g), Neomycin (N, 30 *μ*g), kanamycin (K, 30 *μ*g), penicillin (P, 6 *μ*g), and vancomycin (VA, 30 *μ*g).

The inhibitory circles emerging after 24 h of incubation were measured. Activity was assessed as sensitive (≤21 mm), intermediate (16–20 mm), and resistant (≥15 mm), as previously described by Liasi et al. [37].

### 2.6. Acidity and Growth Kinetics in Pure and Mixed Cultures

To study the growth kinetics of* L. mesenteroides* in pure cultures and cultures mixed with pathogens strains (*L. innocua, L. ivanovii*,* and S. aureus*), strain B7 was selected and inoculated by streaking on solid MRS medium and incubated at 30°C for 18 h. After incubation, a colony was inoculated into MRS liquid and was incubated at 30°C for 18 hours. Then, 100 mL of the 18 h culture was inoculated into 10 mL of skimmed milk containing 0.3% yeast extract and was incubated at 30°C for 18 h [38].

The bacterial population measurement with the indicator strains in pure and mixed cultures was performed by counting in Nutrient Agar medium (Oxoid) to differentiate between the colonies of* Leuconostoc* and* Listeria* spp. where latter appear larger, the Baird Parker medium (Oxoid) to count* S. aureus* and MRS to count the* Leuconostoc* strain.

Strain B7, which is the most efficient producer of antimicrobial substances, and three indicator strains, that is, *L. innocua* ATCC 33090*, L. ivanovii* ATCC 19119 and* S. aureus* ATCC 43300, were routinely subcultured in 10 mL of skim milk with 0.3% yeast extract (Oxoid) that had an initial concentration of 10^7^ CFU/mL for* L. innocua* ATCC 33090, 10^7^ CFU/mL for* L. ivanovii* ATCC 19119, and 10^7^ UFC/mL for* S. aureus* 43300. The three strains were inoculated separately into 100 mL of skim milk for monitoring pure cultures, and the mixed culture was prepared by mixing a culture of the indicator strains with the test strain B7 at a concentration of 10^7^ CFU. The cultures were divided into tubes and incubated at 30°C for 24 h. Every three hours, the samples were aseptically withdrawn from tubes to determine the pH, titrable acidity, and the growth rate. This experiment was repeated three times [4].

### 2.7. Statistical Analysis

Several statistical models have been proposed to estimate growth parameters from the curves obtained by the counting methods. An ANOVA and range tests were used to evaluate the difference between the average of pH, acidity, and bacterial load and were represented by log *N* and *μ*max. The significance of the variation in the results of the antimicrobial activity was evaluated by two factors: repeatability and reproducibility [39, 40].

## 3. Results

### 3.1. Isolation, Selection, and Identification of *Leuconostoc* Isolates from Raw Camel Milk

A total of thirteen* Leuconostoc* strains were isolated from camel milk. The isolates exhibited ovoid shape and were associated in short pairs and/or chains. All isolates were Gram-positive, catalase negative, citrate positive, able to produce CO_2_ from glucose, able to produce dextran from sucrose, and unable to hydrolyse arginine. According to the antibacterial test, B7 and Z8 showed more inhibition zones than the other strains. Therefore, these two strains were selected for probiotic profiling and behavioural studies in the presence of food borne pathogens. The results obtained from physiological fermentation profiling (Table 1) and 16 srRNA as a molecular technique (data not shown) identified B7 and Z8 as* L. mesenteroides* and revealed 99% homology with other sequences from the reference strains deposited in the GenBank, according to the BLAST tool.

### 3.2. Genetic Identification of the Bacteriocin

**Figure 1:**
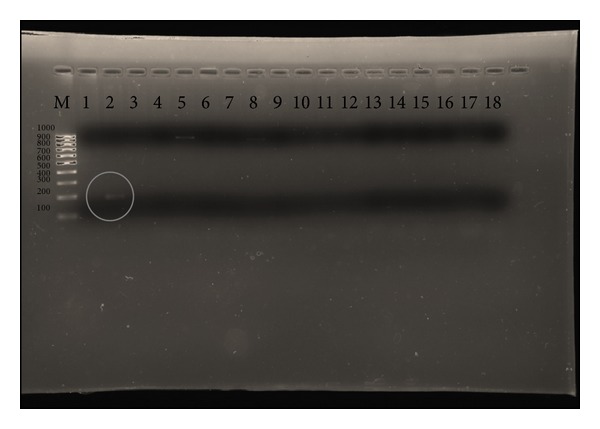
The amplification of bacteriocins produced by *Leuconostoc mesenteroides* B7 strain with LcnB primers; lane M, MW marker; lane 2, *Leuconostoc mesenteroides* B7B; lane 5, *Leuconostoc mesenteroides* B7′B; and lane 8, *Leuconostoc mesenteroides* RB.

Specific primers for mesenterocin Y, mesB and Leucocin A, LcnB, and LcnA-TAF were tested on the extracted DNA. As shown in Figure 1, the specific primer for leucocin B produced a faint PCR product of the expected molecular weight. Sequencing and alignment with respect to sequences deposited in GenBank database confirmed matching with respect to a bacteriocin (mesY, mesC, mesD, mesE, mesF, mesH, and mesB) from* L. mesenteroides* (data not shown).

### 3.3. Antimicrobial Activity of *Leuconostoc* Isolates

**Figure 2:**
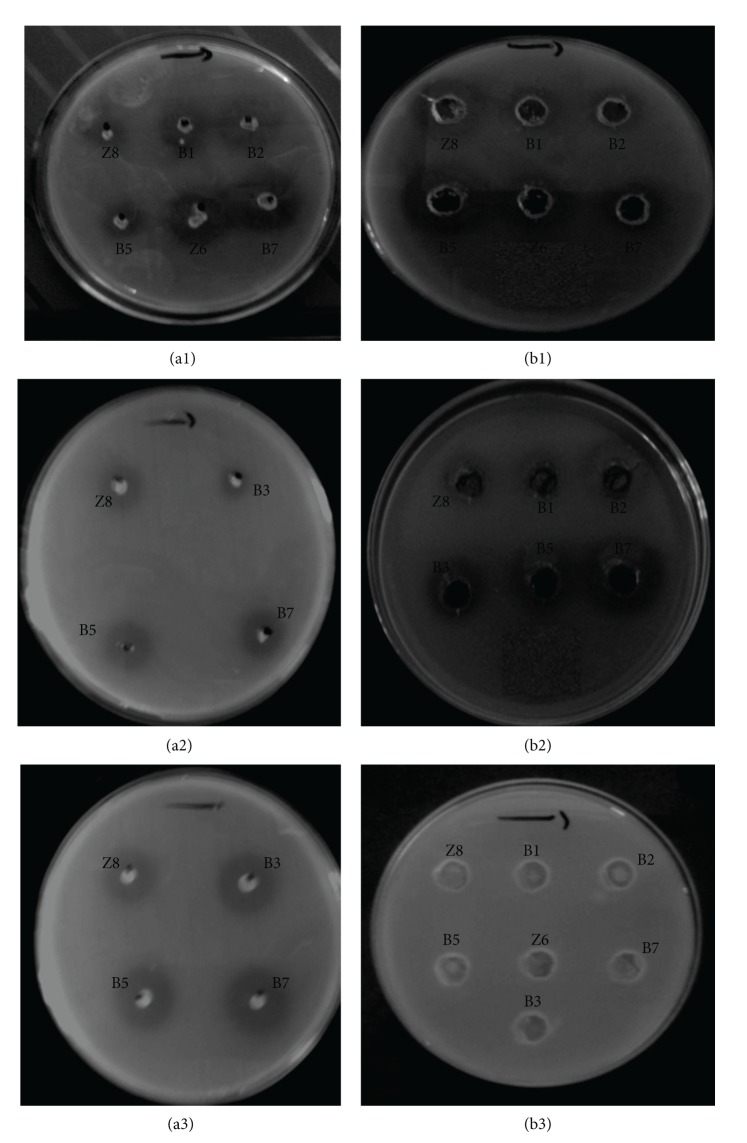
The inhibition spectra of *Leuconostoc mesenteroides* strains against indicator microorganisms; B1, B2, B5, B7, and B3: *Leuconostoc mesenteroides* isolated from camel milk sample 1; Z6 and Z8: *Leuconostoc mesenteroides* isolated from camel milk sample 2. (a1) Inhibition of *Listeria innocua* (ATCC 33090) by *Leuconostoc mesenteroides* using a direct method. (a2) Inhibition of *Listeria ivanovii* (ATCC 19119) by *Leuconostoc mesenteroides* using a direct method. (a3) Inhibition of *Staphylococcus aureus* by *Leuconostoc mesenteroides* using a direct method. (b1) Antibacterial activity of *Leuconostoc mesenteroides* versus *Listeria innocua* (ATCC 33090) using a buffered medium. (b2) Antibacterial activity of *Leuconostoc mesenteroides* versus *Listeria ivanovii* (ATCC 19119) using a buffered medium. (b3) Antibacterial activity of *Leuconostoc* versus *Listeria ivanovii* (ATCC 19119) using a buffered medium treated by chymotrypsin.

B7 and Z8 strains exhibited inhibitory activity against several pathogenic bacteria, including* S. aureus*: 43300,* L. innocua* (ATCC 33090), and* L. ivanovii* (ATCC 19119). The inhibition zones were measured, and the results indicated that the inhibition intensity and range varied depending on the* Leuconostoc* species assayed (Figure 2). Furthermore, to investigate whether the cause of the inhibition was due to the protein substance, buffered supernatants adjusted to pH 6.8 were treated with proteolytic enzymes, which led to the disappearance of the inhibition zones (Figure 2). This result indicated that inhibition was caused by a proteinaceous compound.

However, inhibition remained after heating the bacterial supernatants to a temperature of 100°C (data not shown), which indicated that the causative inhibitory agent is heat resistant. These results agree with previously reported results [41–45].

### 3.4. pH Tolerance

The viable cell counts of the two* L. mesenteroides* strains after a 3 h exposure to low pH are shown in Table 2. Strain B7 was viable at all pH levels. The results showed a decrease of 21.17% at pH 2 but increases of 0.49% and 5.06% at pH 3 and 4, respectively. The* L. mesenteroides* strain Z8 was not viable at pH 2, but marked increases of  7.49% and 2.43% were observed at pH 3 and 4, respectively. The highest viability was observed at pH 4.

### 3.5. Bile Salt Tolerance and Bile Salt Hydrolysis

The results of bile salt tolerance assay are shown in Table 3. The two* L. mesenteroides* strains were able to grow in the presence of 0.5%, 1%, and 2% oxgall. The reduction ranges after a 4 h exposure were 18.21%–21.27% and 1.92%–13.53% for strains B7 and Z8, respectively. The highest resistance was observed in B7. Neither* L. mesenteroides* strain was able to hydrolyse bile salt.

### 3.6. Resistance to Pepsin

Neither strain was able to survive at pH 2 when 3 mg/mL of pepsin was added. However, a remarkable resistance was observed at pH 3 when 3 mg/mL of pepsin was added. Strain B7 decreased by 2.6%, and strain Z8 decreased by 5.4%. The results are shown in Table 4.

### 3.7. Haemolytic Activity

Neither of the* Leuconostoc mesenteroides* strains was able to hydrolyse human blood, indicating that these strains are nonhaemolytic bacteria.

### 3.8. Antibiotic Sensitivity Test

The diameters of the inhibition zones (in mm) of the antibiotic tested against* L. mesenteroides* strains are shown in Table 5. The two strains were resistant to kanamycin, streptomycin, tetracycline, and vancomycin and were sensitive to amoxicillin, ampicillin, cephalotin, chloramphenicol, erythromycin, lincomycin, and penicillin. A moderate resistance was observed against gentamycin and neomycin.

### 3.9. Kinetic Monitoring of pH Evolution and Acidity

**Figure 3:**
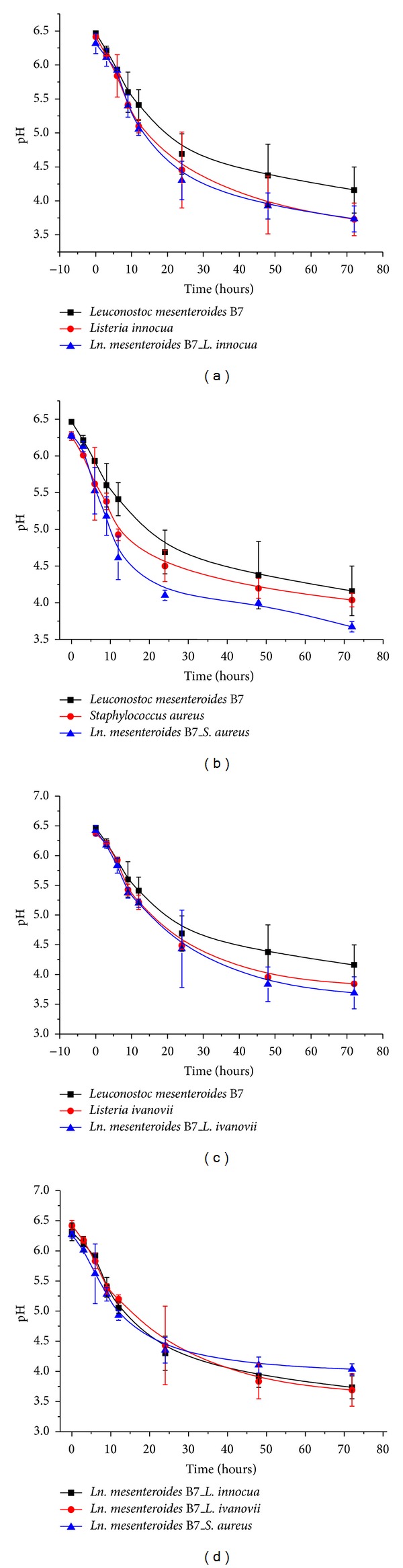
Monitoring the pH of pure and mixed cultures of B7 strain and indicator pathogens. (a) pH variation of *Leuconostoc mesenteroides* B7 and *Listeria innocua* strains in both pure and mixed cultures. (b) pH variation of *Leuconostoc mesenteroides* B7 and *Staphylococcus aureus* strains in both pure and mixed cultures. (c) pH variation of *Leuconostoc mesenteroides* B7 and *Listeria ivanovii* strains in both pure and mixed cultures. (d) pH variation comparison of *Leuconostoc mesenteroides* B7, *Listeria innocua*, *Staphylococcus aureus*, and *Listeria ivanovii* strains in mixed culture.

**Figure 4:**
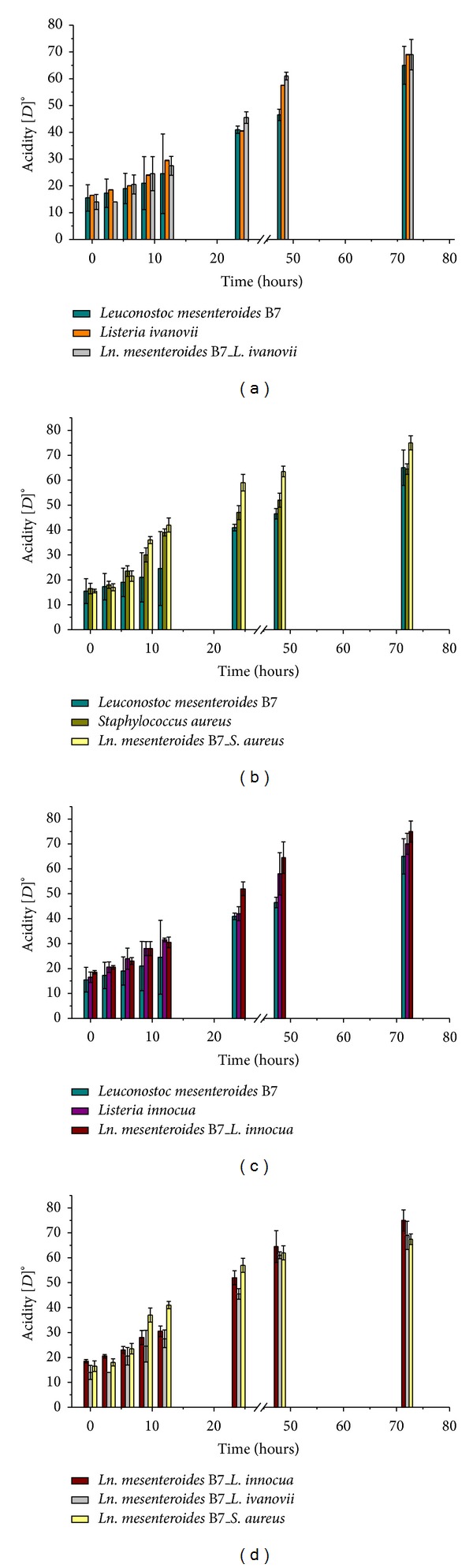
The acidity kinetics of *Leuconostoc mesenteroides* B7 strain and indicator pathogens in pure and mixed cultures. (a) Acidity variation expressed in dornic degree for *Leuconostoc mesenteroides* B7 and *Listeria ivanovii *in both pure and mixed cultures. (b) Acidity variation expressed in dornic degree for *Leuconostoc mesenteroides* B7 and *Staphylococcus aureus* in both pure and mixed cultures. (c) Acidity variation expressed in dornic degree for *Leuconostoc mesenteroides* B7 and *Listeria innocua *in both pure and mixed cultures. (d) Comparison of acidity variation expressed in dornic degree for *Leuconostoc mesenteroides* B7 *Listeria ivanovii, Staphylococcus aureus*, and *Listeria innocua*, respectively, in mixed culture.

The evolution of pH in pure and mixed cultures can be observed in Figures 3 and 4. A significant pH decrease was observed in the mixed cultures after 72 h for the three indicator strains. Thus, *L. innocua*, *L. ivanovii*, and *S. aureus* pure cultures were less acidifying in milk medium as compared to the mixed cultures. Accordingly, significant pH decreases from 6.41 ± 0.01 to 3.73 ± 0.24 for *L. innocua*, from 6.27 ± 0.03 to 3.87 ± 0 for *L. ivanovii*, and from 6.37 ± 0.00 to 3.84 ± 0.03 for *S. aureus* were determined.

### 3.10. The Growth Kinetics of Pathogenic Indicator Strains Pure Cultures and Cultures Mixed with a *Leuconostoc* Strain

**Figure 5:**
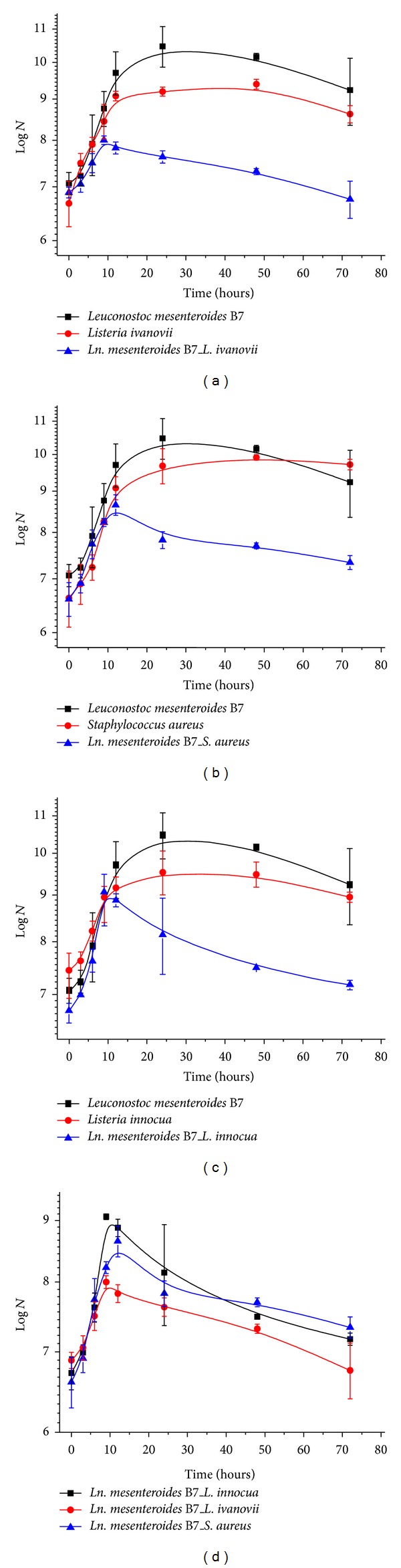
The growth kinetics of *Leuconostoc mesenteroides* B7 strain and indicator pathogens in pure and mixed cultures. (a) Graphical representation of the growth kinetics expressed in log *N* of *Leuconostoc mesenteroides B7* and *Listeria ivanovii* in pure and mixed cultures. (b) Graphical representation of the growth kinetics expressed in log *N* of *Leuconostoc mesenteroides B7* and *Staphylococcus aureus* in pure and mixed cultures. (c) Graphical representation of the growth kinetics expressed in log *N* of *Leuconostoc mesenteroides B7* and *Listeria innocua* in pure and mixed cultures. (d) Graphical representation of the growth kinetics expressed in log *N* of *Leuconostoc mesenteroides B7*, *Listeria ivanovii, Staphylococcus aureus*, and *Listeria innocua*, respectively, in mixed culture.

The maximum growth rate “*μ*max” was estimated using the model described by Baranyi and Roberts [39]. Significant reductions of the listerial load after the addition of the B7 protective culture were observed, as shown by a regression in the G time. The largest bacterial regression was attributed to* Listeria innocua* ATCC 33090 (Figure 5). A lower decrease in the staphylococcal load was also observed after the addition of strain B7.

The maximum growth rate (*μ*max) of the control* L. innocua *ATCC 33090 culture was 0.243. The *μ*max in the presence of strain B7 was 0.148, which is a growth delay of 109.2 min compared to the control (Figure 5).* Listeria ivanovii* ATCC 19119 exhibited a *μ*max of 0.219 in pure culture and 0.168 in mixed culture, which is a growth delay of 57.6 min. The *μ*max of *S. aureus* ATCC 43300 was 0.338 for the control and 0.293 in the presence of strain B7 with a growth delay of 31 min.

Following the study of the growth kinetics, culture B7 showed relatively a slow growth. Bioprotective strains promoted their own growth to control pathogens by inhibiting pathogenic growth. The reductions in the listerial loads were approximately 1.87 and 1.78 log units for* L. innocua* and* L. ivanovii*, respectively. A reduction of approximately 1.38 log units was observed for* S. aureus*, which was a smaller reduction than those observed for* Listeria* strains.

## 4. Discussion

Two* Leuconostoc* strains (B7 and Z8) isolated from camel milk were characterised by their genetic profile, probiotic profile, and behaviour against food-borne pathogens in mixed culture. The antimicrobial activities exhibited by these strains were sensitive to proteolytic enzymes but were heat stable; therefore, the antimicrobial activity may be due to heat-stable protein or peptides.

In the present study, the genes responsible for the production of bacteriocin were detected using LcnB primers. Interestingly,* L. mesenteroides* B7 showed the expected molecular weight for a leucocin B, suggesting that these strains should be examined on the genetic and functional levels. The molecular characterisation by the 16s rRNA gene was in agreement with the phenotypic characterisation. The strains exhibited high similarity among themselves and with sequences from the reference strains in GenBank.

Probiotic foods should maintain the viability of the probiotic bacteria during the preparation and shelf life of the products and during the transit through the gastrointestinal tract to exert their beneficial effects [44]. Selecting potential probiotic strains that can effectively perform in the gastrointestinal (GI) tract is a significant challenge [45]. Therefore, we characterised the probiotic profiles of two strains of* L. mesenteroides* (B7 and Z8). Acid and bile tolerance were two fundamental properties that indicate the ability of these microorganisms to survive through the host GI tract [46].

Argyri et al. [47] found no resistance to low pH for 16* L. mesenteroides* strains from a total of 17. One strain was able to resist exposure to pH 2.5, but the viable counts of most strains were less than 1 log CFU/mL after 3 h. Our results showed a good viability for the two *L. mesenteroides* when exposed to the acidic condition of the stomach (pH 3 and 4). The viable count of strain B7 in pH 2 was 6.59 log CFU/mL, but strain Z8 showed no viability.

The presence of bile salt in the small intestine is another challenge for probiotic bacteria. The two* L. mesenteroides* strains survived well in the presence of different concentrations of bile salt (0.5, 1, and 2% (w/v)), with some loss in viability. A recent study showed that bile salt affected the growth rate and ability of isolated* L. mesenteroides* subsp. *mesenteroides* [18]. Surono [48] found that* L. mesenteroides* subsp.* mesenteroides* IS-27526 had a poor survival rate of 4.37 log CFU/mL in the presence of 0.3% oxgall (w/v). Allameh et al. [18] showed the tolerance of* L. mesenteroides* subsp.* mesenteroides* after 2, 4, and 8 h incubation periods in presence of 0.0, 0.15, and 0.3% of bile salt, respectively. The results of this study showed not only viability but also proliferation in all three concentrations for all incubation periods.

A probiotic needs to survive conditions such as low pH, pepsin, and pancreatin activity and bile while adhering to epithelial cells and competitively excluding pathogens [49]. In our study,* L. mesenteroides* strain B7 was viable at pH 2, but neither strain showed viability at pH 2 when 3 mg/mL of pepsin was added. However, both strains were viable at pH 3 and 4 with pepsin supplementation. A similar study by Seo et al. [50] showed that* L. mesenteroides* YML003 exhibited a higher survival of 1 · 7 · 10^5^ CFU/mL after exposure to artificial gastric juices, with an initial cell number of 2 · 5 · 10^8^ CFU/mL.

The absence of haemolytic activity and antibiotic resistance are considered safety prerequisites for the selection of a probiotic strain [51]. No zones were detected around the colonies of the two* L. mesenteroides* strains when grown in Columbia human blood agar, suggesting that there was no *γ*-haemolytic activity* in vitro*. The lack of *γ*-haemolytic activity is a desirable trait in probiotic bacteria. Several authors have shown similar results [18–47].

In addition, the two strains were resistant to kanamycin, streptomycin, tetracycline, and vancomycin but were sensitive to amoxicillin, ampicillin, cephalotin, chloramphenicol, erythromycin, lincomycin, and penicillin. Moderate resistance was observed against gentamycin and neomycin. Our results agree with those obtained in previous studies; the observed sensitivity to ampicillin, cephalotin, erythromycin, lincomycin, and penicillin and resistance to vancomycin are similar to results obtained by Zarour et al. [52], and sensitivity to chloramphenicol and ampicillin was also observed by Allameh et al. [18]. All studies showed resistance to vancomycin in* L. mesenteroides* strains. Vancomycin resistance is a general intrinsic feature that is linked to the presence of a pentadepsipeptide with a C terminal-lactate instead of a d-alanine in the peptidoglycan [53]. Few reports are available on other antibiotics.

Our* in vitro* studies demonstrated that the two* L. mesenteroides* strains had good probiotic profiles. These strains exhibited high viability at low pH levels, both in the presence of 2% of bile salt and in the presence of pepsin. These strains have acceptable susceptibility antibiotic profiles and are nonhaemolytic bacteria.* L. mesenteroides* strains B7 and Z8 could be ideal probiotic candidates.

These interactions can be the stimulation of one or more microorganism or may correspond to the inhibition of growth or metabolic activity. Inhibition may occur through the production of inhibitory substances or when one of the two microorganisms is inhibited by another. Inhibition could also be induced by reciprocal competition [54, 55]. Therefore, to study the behaviour of these two strains against food-borne pathogens, a kinetic profile of these strains was measured in both pure and mixed cultures.

Monitoring the pH and acidity showed a significant variability between pure and mixed culture, a result that can be explained by the production of organic acids (lactic and acetic acids). Therefore, we can conclude that the incubation time positively influenced the performance of the* L. mesenteroides* strains. Consequently, we can see that the amount of acid produced varies depending on the life stage of the bacterium.

The growth curve analysis in the mixed cultures showed a significant reduction of pathogen bacteria growth after 9 h of incubation during the late exponential phase of growth, which can explain the inhibition of the B7 strain towards *Listeria* and *Staphylococcus* through the production of inhibitory substances such as bacteriocins. Similar results were reported by Lacroix and Millette [56]. The antimicrobial activity of bacteriocin-producing LAB against pathogens was explained by the production of bacteriocins in broth cultures, which was estimated to be maximal after 9 hours of incubation, where the maximum number of bacteria had been attained in the early stationary phase of growth.

Study of the antimicrobial activity against* L. ivanovii* ATCC 19119 showed a small variation of repeatability (giving a lower limit of the variability of results), which indicates an internal dispersion close to the results in homogeneous coefficients of repeatability (1.54%, 1.71%, and 1.76%) (Table 6). Conversely, the pathogen strains showed variability in their growth rates, meaning that the observed potential showed an inhomogeneous distribution indicated by a coefficient of reproducibility of 3.48% (Table 6). The estimation of this activity against* S. aureus* ATCC 43300 displayed a minor, insignificant variability in the coefficients of repeatability (1.60%, 2.65%, and 0.82%) (Table 6), which is explained by a lower antistaphylococcal capacity than the antilisterial capacity of* L. mesenteroides* B7.

## 5. Conclusion

In conclusion, the results of this study showed that the two strains of* Leuconostoc mesenteroides* (B7 and Z8) were found to possess good probiotic properties* in vitro*.   Moreover, the kinetic studies showed that these two strains, especially B7, can be used as protective cultures to inhibit pathogenic bacteria growth in food. Therefore, these strains are good candidates for further investigation with* in vivo* studies to elucidate their potential health benefits and in fermentation studies to assess their technological characteristics for applications as novel probiotic starters.

## Figures and Tables

**Table 1:** Physiological tests and fermentation profiling of bioactive *Leuconostoc* strains isolated from camel milk.

Strains	Catalase	Growth in the presence of NaCl		pH		Growth at					Fermentation profile											
		3%	6.5%	4.8	6.8	4°C	15°C	30°C	37°C	45°C	Arabinose	Maltose	Rhamnose	Esculin	Manitol	Sorbitol	Galactose	Lactose	Fructose	Glucose	Sucrose	Xylose
Z8	−	+	−	−	+	−	+	+	+	−	−	+	−	−	−	−	+	+	+	+	+	+
B7	−	+	−	−	+	−	+	+	+	−	−	+	−	−	−	−	+	+	+	+	+	−

**Table 2:** The effect of low pH on the viability of *Leuconostoc mesenteroides* strains.

	pH2		pH3		pH4	
	0 h	3 h	0 h	3 h	0 h	3 h
Ln. B7	8.36 ± 0.29	6.59	8.16	8.20	8.29 ± 0.14	7.87
Ln. Z8	7.99	0	8.14 ± 0.007	8.75 ± 0.007	8.62 ± 0.007	8.83 ± 0.007

All results are expressed as log CFU/mL. Values in the same row followed by a different letter are significantly different (*P* < 0.05).

Ln. B7 refer to *Leuconostoc mesenteroides* B7.

Ln. Z8 refer to *Leuconostoc mesenteroides* Z8.

**Table 3:** The effect of oxgall concentration on the viability of *Leuconostoc mesenteroides* strains.

	0.5%		1%		2%	
	0 h	4 h	0 h	4 h	0 h	4 h
Ln. B7	8.45 ± 0.007	6.86 ± 0.14	8.65 ± 0.19	6.81 ± 0.18	8.15 ± 0.007	6.66
Ln. Z8	8.35 ± 0.056	7.22 ± 0.16	8.30 ± 0.056	7.92 ± 0.056	8.33 ± 0.007	8.17 ± 0.23

All results are expressed as log CFU/mL. Values in the same row followed by a different letter are significantly different (*P* < 0.05).

Ln. B7 refer to *Leuconostoc mesenteroides* B7.

Ln. Z8 refer to *Leuconostoc mesenteroides* Z8.

**Table 4:** The effect of pepsin on the viability of *Leuconostoc mesenteroides* strains.

	Pepsin (pH2)		Pepsin (pH3)	
	0 h	3 h	0 h	3 h
Ln. B7	7.18	0	7.71	7.51
Ln. Z8	8.81	0	8.72 ± 0.071	8.25

All results are expressed as log CFU/mL. Values in the same row followed by a different letter are significantly different (*P* < 0.05).

Ln. B7 refer to *Leuconostoc mesenteroides* B7.

Ln. Z8 refer to *Leuconostoc mesenteroides* Z8.

**Table 5:** Antibiotic susceptibility of *Leuconostoc mesenteroides* strains.

Antibiotics	Symbol	*μ*g/disc	Clear zone diameter (mm)			
			Z B7		Z8	
Amoxicillin	AMX	25	25	S	25	S
Ampicillin	AM	10	23	S	24	S
Céphalotin	CEF	30	23	S	24	S
Chloramphenicol	C	30	28	S	27	S
Erythromycin	E	15	27	S	28	S
Gentamycin	GM	10	18	I	18	I
Kanamycin	K	30	12	R	13	R
Lincomycin	L	15	25	S	25	S
Neomycin	N	30	16	I	16	I
Penicillin	P	6	23	S	24	S
Streptomycin	S	10	14	R	15	R
Tetracycline	TE	30	14	R	14	R
Vancomycin	VA	30	00	R	00	R

R: resistance, I: intermediate, and S: sensitive.

**Table 6:** Statistical analysis of the growth kinetics (variance analysis).

Variance analysis							
	*L. ivanovii *	*L. innocua *	*St. aureus *		*L. ivanovii *	*L. innocua *	*St. aureus *
Test number *P*	2	2	2	Repeatability variance *σ*_*r*_^2^	0.091	0.144	0.161
Result number *N*	20	20	20	cv repeatability	1.20%	2.17%	1.17%
Standard deviation	0.098	0.049	0.091	Reproducibility variance *σ*_*R*_^2^	0.22	0.441	2.673
Variance	0.009	0.002	0.008	cv reproducibility	3.42%	7.54%	13.52%

Mean (Log *N*)							
8.00	8.065	8.22					

SD, Var, and mean at 9 h of incubation.
